# An Efficient and Effective Model to Handle Missing Data in Classification

**DOI:** 10.1155/2020/8810143

**Published:** 2020-11-25

**Authors:** Kamran Mehrabani-Zeinabad, Marziyeh Doostfatemeh, Seyyed Mohammad Taghi Ayatollahi

**Affiliations:** Department of Biostatistics, Faculty of Medicine, Shiraz University of Medical Sciences, Shiraz, Iran

## Abstract

Missing data is one of the most important causes in reduction of classification accuracy. Many real datasets suffer from missing values, especially in medical sciences. Imputation is a common way to deal with incomplete datasets. There are various imputation methods that can be applied, and the choice of the best method depends on the dataset conditions such as sample size, missing percent, and missing mechanism. Therefore, the better solution is to classify incomplete datasets without imputation and without any loss of information. The structure of the “Bayesian additive regression trees” (BART) model is improved with the “Missingness Incorporated in Attributes” approach to solve its inefficiency in handling the missingness problem. Implementation of MIA-within-BART is named “BART.m”. As the abilities of BART.m are not investigated in classification of incomplete datasets, this simulation-based study aimed to provide such resource. The results indicate that BART.m can be used even for datasets with 90 missing present and more importantly, it diagnoses the irrelevant variables and removes them by its own. BART.m outperforms common models for classification with incomplete data, according to accuracy and computational time. Based on the revealed properties, it can be said that BART.m is a high accuracy model in classification of incomplete datasets which avoids any assumptions and preprocess steps.

## 1. Introduction

One of the most widely used areas of data mining is prediction [1]. When the subject of prediction is assignment of individuals into the groups, the prediction is called classification [2]. Classification in medical sciences is very vital as it is a matter of life or death [3]. With the accurate classification, illness or even death can be prevented; therefore, in addition to avoiding wasting medical resources, life expectancy is increased [4].

In classification, sample information can be used to identify high-risk people or even to specify the stage of a disease [5]. There are several models for classification which are carried out via statistical modeling and/or learning algorithms such as logistic regression, decision tree, random forest [6], and naïve Bayes. One of the most applicable supervised learning methods is the decision tree. Each tree includes a set of logical rules for the independent variables (node). Each branch of the tree goes as far as a leaf. The leaves of the classification tree are corresponded to one of the response levels [7].

The decision tree model has a simple and understandable logic, although research has shown that ensemble of single trees together and combining the results increase the accuracy of the classification [8]. The most well-known sum of the tree model is the random forest [6]. This model, due to its strong classification accuracy, has attracted a lot of attention and has been widely used in various fields of science. It builds multiple classification trees where the growth of each tree is independent of the other trees. To classify a new observation, the majority vote of the random forest trees is used as the final classification level [6].

Machine learning algorithms are not directly based on statistical models. Chipman et al. in 2010 [9] introduced a combined machine learning and statistical model named “Bayesian additive regression trees” (BART), which is a combination of trees where the statistical Bayesian model provides regularization of each tree. In recent years, BART has become popular in numerous areas as a great prediction model [10–14], more specifically in medicine [15–19]. Therefore, in this study, the BART model was chosen as the classification model.

Although classification models have been developed and newer models with higher accuracy have been proposed, some items reduce the classification accuracy. One of these items is the presence of the variables which have no effect on the response [20, 21]; hence, a variable selection step is required before use of classification models. Another item that has a negative effect on classification models is the presence of missing values [1, 22, 23]. This is despite the fact that in many studies, specifically in the field of medical sciences, missingness becomes inevitable [24–26]. Therefore, there is a need for classification models to be more robust in terms of incomplete datasets to keep a high accuracy in the presence of missing data.

There are different ways to deal with datasets with incomplete cases. The simplest way is the list-wise deletion method; in this method, any case that has only one missing value in their variables is completely excluded from the dataset. It is clear that this method causes loss of information, which can cause reduction in classification accuracy [27, 28]. A common and more complex approach facing missingness is imputing the missing values. Various methods are available for imputation, but each one has their own advantages and disadvantages [27, 29]. Since there is no unique method for imputation to have the best action in all conditions, in practice, one has to pay attention to the characteristics of each data set and the missingness pattern to choose the best imputation method.

For handling missing data in a classification field, there are approaches which handle missing data without any imputation or loss of information [30, 31]. As these approaches do not face selection of the proper imputation method, they are much more user-friendly and practical.

Another inefficiency of methods that handle missing data in the classification field is that most of these methods solely handle missing values in the train step and cannot classify new data with missing values unless with separate imputation [23]. This way, in the training step, first of all, the missing values should be imputed, and then the model will be trained. However, the trained model cannot be run on a data that have missing values. Therefore, to classify these new data, there is a need of another imputation; then, the problems of imputation such as proper sample size or selection of the proper imputation method arise again. Since in real-world classification applications, it is very likely to have data sets where at least one of its variables has a missing value, and a method that can perform the classification in the presence of missing values is more appropriate and practical.

“Missingness incorporated in attributes” (MIA) [32] is an approach that natively manages missing data in decision trees in a way that new data with missing values can be predicted without any loss of information or even imputation. Kapelner and Bleich presented a model which enhanced BART with MIA and investigated its properties in regression [33].

This research aims at introducing the implementation of MIA within BART for binary classification, which is applicable to classify datasets with missing values with no need for imputing in the training step or new data classification and no need to drop incomplete cases. This model was performed on simulated and real data to investigate its strength on different scenarios and fields. The expectation is that BART.m brings more flexibility to deal with incomplete datasets and provides a higher classification accuracy compared to BART and random forest models which run on datasets completed by imputation.

## 2. Materials and Methods

The BART model gained both statistical Bayesian inference and machine learning strengths [9]. For the binary outcome, the BART model is presented as a probit model in equation [(1)].

(1) $$\begin{array}{c} P\left(Y=1\;|\;x\right)=\Phi \left[{Τ}_{1}^{{Μ}_{1}}\left(X\right)+{Τ}_{2}^{{Μ}_{1}}\left(X\right)+\cdots +{Τ}_{m}^{{Μ}_{m}}\left(X\right)\right], \end{array}$$

Where *Y* is the binary response, *Τ*_*j*_ denote the *j*-th tree structure of *m* distinct trees, *X* is the predictors, and *Μ*_*j*_ represents parameters in leaves. Finally, *Φ* is the cumulative distributive function of the standard normal distribution.

The use of Bayesian priors provides an approach to regularization which causes higher classification accuracy. Priors in classification have two components, the first one controls the node's depth which limits the complexity of any single tree; the second one shrinks the leaf parameters toward the center of the response distribution. These priors provide a strategy to avoid overfitting by allowing the data to speak more naturally. The “bartMachine” Package [34] in *R* language [35] was used to run the BART model.

BART, as mentioned earlier, is a good model for prediction but cannot handle data with missing values [33]. MIA is an approach that could be implemented into the BART structure to handle missing data. MIA does not require any assumptions or need for imputation; it modifies the tree splitting rules during model construction. The procedure is described in the following.

**Figure 1:**
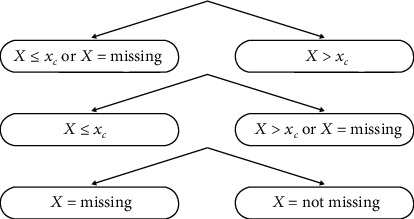
Splitting rule choices of nodes in MIA.

Each node of the decision tree investigates which variable is better to classify and then splits the variable in two parts in that node. The MIA procedure adjusts these splitting rules according to Figure 1.

When implementing the rules of MIA during construction of the BART trees, a new model is constructed, which is referred as BART.m [33]. Since BART.m incorporates missing data natively in its structure, it does not require any imputation and can make predictions on future data with missing values [33]. Based on the advantage of BART.m, in this study, the BART.m model is chosen to investigate its properties in the binary classification field.

BART and random forest classification models were used as competing models to compare the BART.m model performance. Hence, these two models cannot run on incomplete datasets, and the datasets become completed via imputation. For imputing incomplete data, it is fair to use the good imputation method; so, the missForest [36] model was chosen as the imputation method. missForest is a time-consuming method but returns very good precision [24, 37, 38], so is the proper method for comparison. To run Random Forest and missForest, the R packages “randomForest” [39] and “missForest” [40] are used, respectively.

To investigate BART.m abilities in different scenarios, both simulated and real data are used. The simulated data was generated under the logistic regression model [2].

(2) $$\begin{array}{c} \text{logit}\left(\frac{p}{1-p}\right)=-0.3+0.7X_{1}+0X_{2}-0.6X_{3}+1.2X_{4}-1X_{1}X_{4}, \end{array}$$

where

(3) $$\begin{array}{c} X_{1}\sim \text{Ber}\left(.5\right)\text{ and }\left[X_{2},X_{3},X_{4}\right]\sim N_{3}\left(\left[\begin{array}{c} 0\\ 0\\ 0 \end{array}\right],\left[\begin{array}{ccc} 1 & .1 & .1\\ .1 & 1 & .1\\ .1 & .1 & 1 \end{array}\right]\right). \end{array}$$

Thus, the simulated binary response is related to one binary independent variable (*X*_1_), one irrelevant variable (*X*_2_), two continuous variables with different effects (*X*_3_ and *X*_4_), and one interaction (*X*_1_*X*_4_). 2000 datasets with sample size of 1000 were generated under model 2; for this purpose, the “SimCorrMix” Package [41] in *R* language was used.

In the next step, in each dataset, missingness was generated under three missing mechanisms [42]:

1. Missing Completely at Random (MCAR)
2. Missing at Random (MAR)
3. Missing Not at Random (MNAR)

In MCAR, all cases have the same probability of being missed; with MAR, the information about the missing data is in the observed data and with MNAR, the information about the missing data is in the missing data itself. These missingness mechanisms were applied in each variable separately. For MCAR and MNAR, the mechanism is obvious, and for MAR, the following mechanism is chosen.

- *X*_1_ becomes miss with the probability depended to *X*_2_
- *X*_2_ becomes miss with the probability depended to *X*_3_
- *X*_3_ becomes miss with the probability depended to *X*_4_
- *X*_4_ becomes miss with the probability depended to *X*_1_

The mentioned missing mechanism was generated by the ampute function of the “mice” package [43] in *R* language.

In addition to the missing mechanism, various missing proportions were also considered. In literature, the upper threshold of missing proportion is 50% [36, 44, 45], but since BART.m does not use any imputation [33], missing proportion up to 90% is used in this study. To have a fair comparison of the missing proportions effects on the classification accuracy, another scenario was considered which is the missing variable that is completely removed from the classification models.

To obtain classification accuracy, the model trained with one dataset was then tested on another dataset with the same missing proportion, and this process was repeated 1000 times.

In addition to simulation, it is important to investigate the effectiveness of the BART.m model on real datasets. For this purpose, ten real-world incomplete two-level classification datasets are selected from the UCI machine learning repository [46].  Table 1 presents the information on these 10 datasets.

It is clear that these datasets cover a wide range of domains and specifications. As these datasets can reflect various problems and applications in real-world datasets, they can provide a good benchmark to investigate and compare the ability of the different classification models.

Tenfold crossvalidation is used to achieve the accuracy. The process of crossvalidation is stochastic; so, it should be repeated to overcome this variation; therefore, each crossvalidation is repeated 100 times independently and finally, the average of these 100 accuracies is reported.

## 3. Results

In this section, simulation results are presented first followed by the real data results.

### 3.1. Simulation Results

Table 2 presents simulated accuracies based on BART.m and the two competing models in different missing proportions. BART.i and RF.i represent the BART and random forest models which run on imputed datasets with the missForest approach. The “Complete” header represents the situation where a dataset is complete (before making missingness), and the “exclude” header presents a situation where a variable with missing values is completely excluded from analysis. Each accuracy in this table is averaged over 1000 iterations of each scenario combination. It is clear that when the data does not have any missing values, the BART.m and BART.i methods are the same and actually are identical to the BART model.

**Figure 2:**
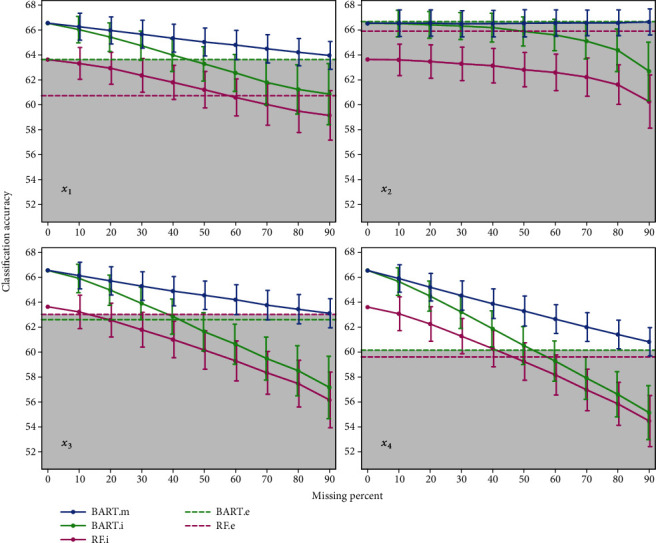
Mean and standard deviation of accuracies achieved by simulation under the MCAR missing mechanism.

For better insights, the plot of Table 2 is shown in Figure 2. Two horizontal dashed lines present the last column of Table 2, where the BART and random forest model run on the dataset where variables with missing values are omitted (denoted by BART.e and RF.e, respectively). Each model that handles missing values is effective up to the point that the model presents higher accuracy than the condition where variables with missing values are excluded from that model. Therefore, in Figure 2, under the upper horizontal line is grey, which indicates any scenario in this grey area that is not suitable.

In Figure 2, it can be seen for the irrelevant variable *X*_2_ that the best result is based on BART.e, which means it is better to not insert *X*_2_ in the classification model; this result was expected but an extraordinary finding is that the BART.m model produces accuracies that are very close to BART.e, which expresses that BART.m can diagnosis irrelevant variables and handle them by its own. For three other variables, the BART.m model is better or the same as BART and random forest models, even for 90 percent missing.

For the discrete variable *X*_1_, the random forest model produces lower accuracy compared to BART.m, such that even for 90% missingness, BART.m has higher accuracy than random forest with complete dataset 0 percent missing values in Table 2 indicate a complete dataset.

It can be seen in Figure 2 that BART.m provides smaller standard deviations for all four variables compared to BART and random forest models. So, in addition to accuracy, BART.m overcomes competing models based on the more reliable results.

By comparing the accuracies of RF.i and BART.i methods in different missing proportions, with the accuracy achieved when the variable with missingness excluded (horizontal lines), it can be concluded that the imputation could be used up to 50% of missingness. Thus, when the missing proportion of a variable is greater than 50%, it is better to remove that variable instead of completing via imputation.

**Figure 3:**
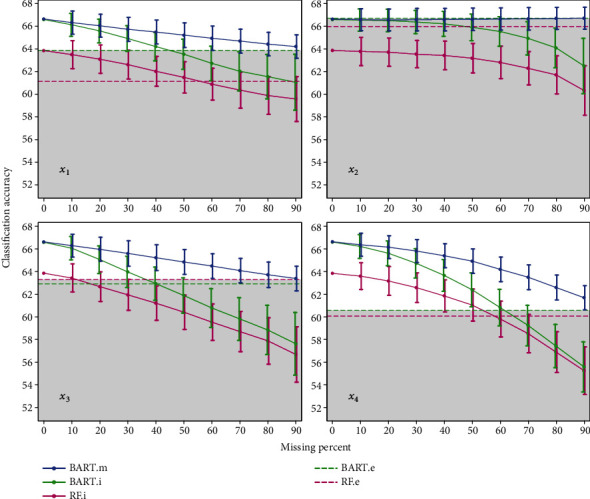
Mean and standard deviation of accuracies achieved by simulation under the MAR missing mechanism.

**Figure 4:**
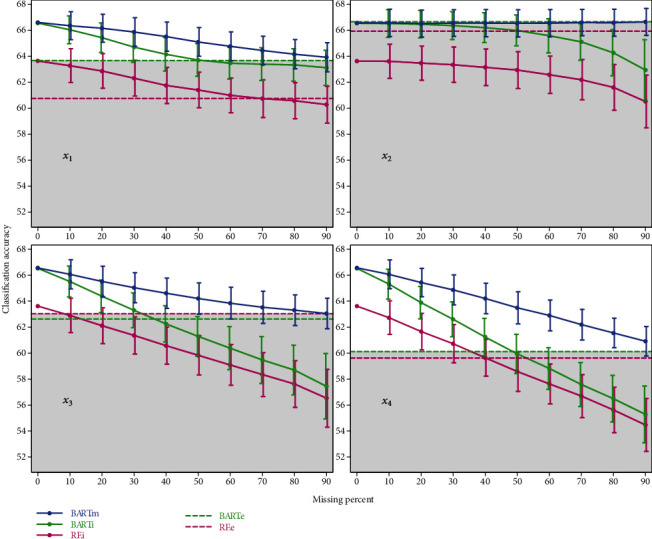
Mean and standard deviation of accuracies achieved by simulation under the MNAR missing mechanism.

The results of MAR and MNAR missing mechanism are not much different from the mentioned MCAR mechanism, tables and figures of these two missing mechanisms are in Tables 3 and 4 and Figures 3 and 4, respectively.

### 3.2. Real Data Results

Table 5 shows the mean and standard deviation of classification accuracies on ten real-world datasets based on BART.m, BART.i, and RF.i methods.

Generally speaking, in real datasets, the accuracy of BART.m is higher or close to competing models. In addition to accuracy, the run time of the classification model is important. The computational time on ten real datasets is presented in Table 6. The implementation was performed on an Intel Core i5 CPU, running at 3.2 GHz and 8 GB RAM. For BART.i and RF.i, the time of the missForest method reported separately to distinguish the required time of the classification algorithm from the imputation procedure. As the training time is usually not the limiting step, the reported time is the computation time to classify unseen instances.

The Ozone Level Detection dataset with 2536 sample size and 73 variables is the biggest dataset in the benchmark (Table 1); BART.m takes less than one minute to run on this dataset but for imputation with missForest, more than four hours is needed. The BART and random forest methods take very low time to classify the complete datasets. By considering the imputation time for BART.i and RF.i, it can be seen that BART.m gained a considerably lower run time.

**Figure 5:**
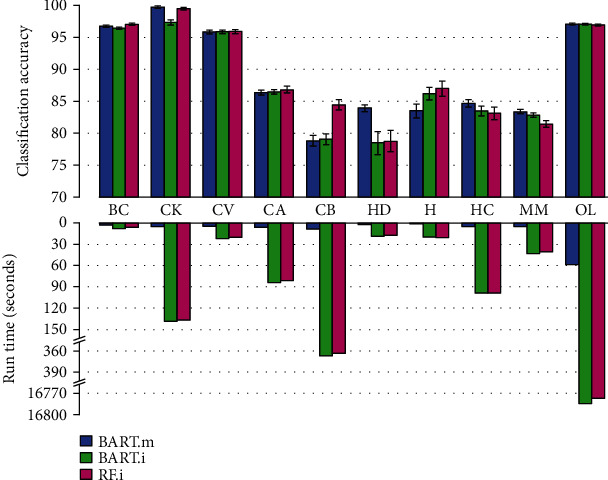
The means and standard deviations of BART.m, BART.i, and RF.i model's classification accuracy (top plot) and corresponding run times per second (bottom plot) on ten real-world datasets. The dataset names are presented as abbreviations in the horizontal axis.

Figure 5 depicts the mean and standard deviation of classification accuracy obtained by BART.m, BART.i (BART+missForest), and RF.i (RF+missForest) methods on ten real-world datasets (Table 5) with their corresponding run time (Table 6).

By checking classification accuracy of real-world datasets next to the corresponding run time in Figure 5, it can be seen that the BART.m model produced accuracies almost as same as the competing models; however, the advantage of BART.m is its low run time.

## 4. Discussion

The BART model is a popular prediction model due to its flexibility and accurate prediction especially in medicine [13, 15–18]. The BART.m model is an extension of BART which can handle datasets with missing values [33]. The properties of BART.m has been investigated in prediction of continuous response [33]; however, to the best of our knowledge, this is the first study that provides the use of the BART.m function in binary classification of incomplete datasets.

The results revealed great capabilities of the BART.m model in binary classification. The simulation findings demonstrate that the BART.m model can diagnose the irrelevant variable for classification and remove its effect. Since the random forest and BART models cannot remove the effect of the irrelevant variables by their own, it is recommended to first use the variable selection step and then remove the irrelevant variables from classification models in order to achieve higher classification accuracy [21]. As BART.m can handle irrelevant variables by its own, the variable selection preprocess is not necessary. This property makes BART.m more flexible.

Both literature [44, 45] and simulation results of this study confirm that imputation is useful up to 50% of missingness. While BART.m does not use imputation, it can be used in missing proportions up to 90%. This great property of BART.m warrants the use of BART.m in any missing proportion, which makes this model more flexible.

There is no universal best imputation method for every situation [27, 29]; hence, for each dataset, the selection of the imputation method is a challenge. Moreover, usually, a good imputation method like missForest is time consuming especially for datasets with higher sample size and more variables [49]. Thus, the BART.m model with no need of imputation becomes a more efficient model.

One of the superiorities of the BART.m model is that it does not require to impute missing values in both training or new data classification steps. So, with BART.m, a new data with missing values can be classified without the need for any extra step. This property of BART.m makes this model accessible because other popular classification models like random forest and BART cannot handle missingness by their own, and they need some preprocess steps like imputation.

Also, the simulation results revealed that for three missing mechanisms MCAR, MAR, and MNAR, the BART.m model outperforms the RF.i and BART.i methods, regardless of discrete or continuous variables and their effects on the response. Moreover, real data finding shows BART.m can produce accuracies close to RF.i and BART.i. As each of the random forest, BART and missForest models are great models, and these results confirm that BART.m can accurately handle missing values in the classification field. This high accuracy classification property of the BART.m model makes this model an effective model in addition to its efficiency.

Yan et al. proposed a Selective Neural Network Ensemble (SNNE) as a classification method to deal with incomplete datasets [31]. They investigated the performance of SNNE on 12 UCI datasets. For all 8 identical datasets with this study, the BART.m's accuracies were very close to those obtained by SNNE. In another study, Tran et al. introduced another approach to handle incomplete datasets in classification [50]. They applied 10 UCI datasets which seven of them had binary response. In the seven aforementioned datasets, BART.m's accuracies were similar to those reported by Tran et al. These findings provide evidence to confirm the effectiveness of the BART.m classification model in dealing with incomplete datasets.

In addition to the valuable properties of BART.m, it should be considered that the model cannot classify incomplete instances when the training dataset is complete; in other words, to classify future instances with missingness, the model should be trained on the incomplete dataset.

For further studies, it is beneficial to investigate the BART.m classification properties in a scenario which multiple variables have missingness.

## 5. Conclusion

This study revealed great capabilities of the BART.m model to classify binary incomplete datasets. As it does not engage to find the best imputation method, it is more practical. It can determine and automatically remove irrelevant variables without any extra task; so, there is no need for a variable selection preprocess step. It can be used even in 90% percent missingness, as well as provide high classification accuracy in just a few seconds. With all of these properties, BART.m becomes a flexible method which can be used by the public without any need of professional knowledge about assumptions and preprocess steps of incomplete classification models. Therefore, BART.m is an efficient and effective model for classification, which has proven to be a working concept incorporating statistical methods in machine learning algorithms.

## Figures and Tables

**Table 1:** Specifications of real-world datasets.

Dataset name	Sample size	Variable number	Discrete variable number	Missing proportion	Imbalance
Breast Cancer Wisconsin [47]	699	10	0	2.29	65.5
Chronic kidney disease	400	24	13	60.5	62.5
Congressional voting records	435	16	16	46.67	61.4
Credit approval	690	15	9	5.36	55.5
Cylinder bands	540	39	19	48.7	57.8
Heart disease—ungarian	294	13	7	99.66	63.9
Hepatitis	155	19	13	48.39	79.4
Horse colic	368	23	15	98.1	63
Mammographic mass [48]	961	5	2	13.63	53.7
Ozone level detection	2536	73	0	27.13	97.1

**Table 2:** Accuracies achieved by simulation under MCAR missing mechanism.

Variable	Model	Complete Variable	Missing Percent									Exclude Variable
			10%	20%	30%	40%	50%	60%	70%	80%	90%	
x_1_	BART.m	66.56	66.27	65.97	65.66	65.34	65.05	64.80	64.50	64.22	63.97	63.65
	BART.i	66.56	66.04	65.44	64.75	63.99	63.29	62.56	61.80	61.24	60.86	63.65
	RF.i	63.63	63.32	62.94	62.36	61.80	61.22	60.60	60.03	59.49	59.15	60.74

x_2_	BART.m	66.56	66.55	66.56	66.50	66.52	66.54	66.56	66.57	66.57	66.65	66.67
	BART.i	66.56	66.53	66.42	66.32	66.20	65.89	65.61	65.11	64.38	62.69	66.67
	RF.i	63.65	63.61	63.47	63.30	63.14	62.82	62.60	62.23	61.62	60.26	65.91

x_3_	BART.m	66.56	66.14	65.71	65.30	64.89	64.56	64.21	63.77	63.44	63.11	62.62
	BART.i	66.56	65.91	64.97	63.90	62.85	61.64	60.64	59.48	58.51	57.16	62.62
	RF.i	63.63	63.23	62.56	61.79	61.01	60.16	59.30	58.34	57.47	56.16	63.03

x_4_	BART.m	66.56	65.91	65.21	64.53	63.88	63.30	62.65	62.01	61.42	60.83	60.14
	BART.i	66.56	65.67	64.49	63.21	61.86	60.52	59.28	57.91	56.61	55.15	60.14
	RF.i	63.63	63.08	62.26	61.28	60.28	59.24	58.17	56.97	55.85	54.47	59.60

**Table 3:** Accuracies achieved by simulation under MAR missing mechanism.

Variable	Model	Complete Variable	Missing Percent									Exclude Variable
			10%	20%	30%	40%	50%	60%	70%	80%	90%	
x_1_	BART.m	66.56	66.26	65.96	65.63	65.37	65.08	64.79	64.52	64.24	64.01	63.65
	BART.i	66.56	66.04	65.47	64.76	64.00	63.28	62.42	61.69	61.17	60.64	63.65
	RF.i	63.63	63.25	62.81	62.31	61.68	61.11	60.47	59.91	59.40	59.07	60.74

x_2_	BART.m	66.56	66.53	66.51	66.54	66.54	66.57	66.60	66.61	66.65	66.67	66.67
	BART.i	66.56	66.50	66.45	66.31	66.14	65.81	65.42	64.78	63.88	62.17	66.67
	RF.i	63.65	63.55	63.48	63.29	63.17	62.90	62.51	61.96	61.34	59.87	65.91

x_3_	BART.m	66.56	66.22	65.87	65.48	65.08	64.68	64.30	63.87	63.48	63.13	62.62
	BART.i	66.56	65.98	64.90	63.74	62.64	61.52	60.34	59.31	58.27	56.98	62.62
	RF.i	63.63	63.17	62.36	61.59	60.81	59.95	59.01	58.13	57.25	55.97	63.03

x_4_	BART.m	66.56	66.30	66.08	65.71	65.27	64.76	63.99	63.25	62.28	61.32	60.14
	BART.i	66.56	66.14	65.50	64.55	63.42	62.06	60.39	58.71	56.77	54.81	60.14
	RF.i	63.63	63.35	62.90	62.27	61.49	60.63	59.32	57.95	56.21	54.46	59.60

**Table 4:** Accuracies achieved by simulation under MNAR missing mechanism.

Variable	Model	Complete Variable	Missing Percent									Exclude Variable
			10%	20%	30%	40%	50%	60%	70%	80%	90%	
x_1_	BART.m	66.56	66.35	66.16	65.87	65.51	65.10	64.76	64.44	64.16	63.92	63.65
	BART.i	66.56	66.04	65.43	64.70	64.15	63.70	63.47	63.39	63.33	63.11	63.65
	RF.i	63.63	63.27	62.86	62.31	61.75	61.40	60.99	60.73	60.57	60.27	60.74

x_2_	BART.m	66.56	66.57	66.52	66.55	66.55	66.54	66.55	66.59	66.58	66.65	66.67
	BART.i	66.56	66.51	66.46	66.36	66.20	65.99	65.57	65.10	64.27	62.93	66.67
	RF.i	63.65	63.62	63.47	63.35	63.14	62.94	62.58	62.19	61.60	60.51	65.91

x_3_	BART.m	66.56	66.07	65.52	65.04	64.61	64.20	63.85	63.53	63.31	63.05	62.62
	BART.i	66.56	65.51	64.38	63.30	62.25	61.30	60.38	59.47	58.70	57.45	62.62
	RF.i	63.63	62.91	62.11	61.36	60.58	59.83	59.09	58.35	57.62	56.53	63.03

x_4_	BART.m	66.56	66.07	65.44	64.87	64.21	63.50	62.90	62.20	61.55	60.92	60.14
	BART.i	66.56	65.32	63.89	62.61	61.24	59.94	58.82	57.58	56.49	55.28	60.14
	RF.i	63.63	62.73	61.66	60.72	59.64	58.59	57.63	56.69	55.63	54.47	59.60

**Table 5:** Mean ± standard deviation of classification accuracies of real-world datasets.

Dataset name	BART.m	BART.i	RF.i
Breast Cancer Wisconsin	96.74 ± 0.20	96.44 ± 0.18	97.06 ± 0.21
Chronic kidney disease	99.76 ± 0.21	97.32 ± 0.43	99.51 ± 0.15
Congressional voting records	95.86 ± 0.30	95.90 ± 0.27	95.92 ± 0.30
Credit approval	86.40 ± 0.40	86.50 ± 0.37	86.85 ± 0.49
Cylinder bands	78.83 ± 0.85	79.08 ± 0.83	84.46 ± 0.76
Heart disease—Hungarian	83.95 ± 0.54	78.47 ± 1.78	78.76 ± 1.63
Hepatitis	83.53 ± 1.11	86.22 ± 0.97	87.00 ± 1.20
Horse colic	84.69 ± 0.57	83.50 ± 0.78	83.15 ± 0.98
Mammographic mass	83.40 ± 0.30	82.82 ± 0.34	81.48 ± 0.52
Ozone level detection	97.10 ± 0.02	97.10 ± 0.05	96.97 ± 0.04

**Table 6:** Run time corresponding to different methods in the application process.

Dataset name	BART.m	missForest	BART	RF
Breast Cancer Wisconsin	0 : 02.44	0 : 05.92	0 : 02.29	0 : 00.04
Chronic kidney disease	0 : 04.71	2 : 16.30	0 : 01.48	0 : 00.05
Congressional voting records	0 : 03.85	0 : 20.17	0 : 01.59	0 : 00.07
Credit approval	0 : 05.99	1 : 21.50	0 : 02.46	0 : 00.11
Cylinder bands	0 : 08.23	6 : 04.30	0 : 02.20	0 : 00.10
Heart disease—Hungarian	0 : 01.98	0 : 17.38	0 : 01.13	0 : 00.05
Hepatitis	0 : 01.45	0 : 19.29	0 : 00.74	0 : 00.05
Horse colic	0 : 05.10	1 : 37.10	0 : 01.44	0 : 00.08
Mammographic mass	0 : 04.58	0 : 40.35	0 : 03.20	0 : 00.12
Ozone level detection	0 : 58.98	4 : 39 : 36.98	0 : 07.81	0 : 00.11

## Data Availability

Ten real-world incomplete two-level classification datasets used to support the findings of this study have been deposited in the UCI machine learning repository ([http://archive.ics.uci.edu/ml]).
